# Do an Altered Gut Microbiota and an Associated Leaky Gut Affect COVID-19 Severity?

**DOI:** 10.1128/mBio.03022-20

**Published:** 2021-01-12

**Authors:** Heenam Stanley Kim

**Affiliations:** a Division of Biosystems & Biomedical Sciences, College of Health Sciences, Korea University, Seoul, Republic of Korea; Rutgers, The State University of New Jersey

**Keywords:** COVID-19, SARS-CoV-2, coronavirus, gut microbiota, gut barrier integrity, leaky gut

## Abstract

Coronavirus disease 2019 (COVID-19), which has been declared a pandemic, has exhibited a wide range of severity worldwide. Although this global variation is largely affected by socio-medical situations in each country, there is also high individual-level variation attributable to elderliness and certain underlying medical conditions, including high blood pressure, diabetes, and obesity. As both elderliness and the aforementioned chronic conditions are often associated with an altered gut microbiota, resulting in disrupted gut barrier integrity, and gut symptoms have consistently been associated with more severe illness in COVID-19 patients, it is possible that dysfunction of the gut as a whole influences COVID-19 severity. This article summarizes the accumulating evidence that supports the hypothesis that an altered gut microbiota and its associated leaky gut may contribute to the onset of gastrointestinal symptoms and occasionally to additional multiorgan complications that may lead to severe illness by allowing leakage of the causative coronavirus into the circulatory system.

## Abstract (precis)

Coronavirus disease 2019 (COVID-19), which has been declared a pandemic, has exhibited a wide range of severity worldwide. Although this global variation is largely affected by socio-medical situations in each country, there is also high individual-level variation attributable to elderliness and certain underlying medical conditions, including high blood pressure, diabetes, and obesity.

## KEY MESSAGES

- While the following remains to be empirically demonstrated, accumulating evidence supports the hypothesis that an altered gut microbiota and an associated leaky gut may contribute to the onset of coronavirus disease 2019 (COVID-19)-related gastrointestinal symptoms, such as diarrhea and, in severe cases, multiorgan complications.
- Testing for a leaky gut and fecal and plasma viral loads may be useful for diagnosing the seriously ill or for preventing transmission by fecal shedding of the virus.
- Fecal microbiota transplantation (FMT), next-generation probiotics focusing on butyrate-producing gut microbes, or simply increasing the daily intake of dietary fiber may be considered in improving the gut health of COVID-19 patients.

## CORONAVIRUS AND THE CORONAVIRUS DISEASE (COVID-19) PANDEMIC

Coronaviruses are enveloped, positive-sense, single-stranded RNA viruses with a genome size of ∼30 kb (1, 2). They are classified into four genera, α, β, γ, and δ, based on their genomes, and α- and β-coronaviruses infect mammals (1). In humans, mild upper respiratory tract infections, such as the common cold, have been reported to be caused by α-coronaviruses (3). However, in the last 2 decades, the world has witnessed three serious outbreaks of more fatal coronavirus diseases in humans, including COVID-19, which has become a pandemic of an unprecedented scale that is pushing health care to its limits worldwide. As of December 2020, over 70 million cases have been reported globally (https://coronavirus.jhu.edu/map.html). The first cases of COVID-19 were reported in 2019 (2). This disease is caused by severe acute respiratory syndrome coronavirus 2 (SARS-CoV-2), which is related to the bat origin β-coronavirus strain SARS-CoV-1, the causative agent of the SARS outbreak of 2002. The SARS outbreak lasted for 2 years and affected 29 countries, resulting in 8,096 cases and 774 deaths (4; https://www.who.int/publications/m/item/summary-of-probable-sars-cases-with-onset-of-illness-from-1-november-2002-to-31-july-2003). Another zoonotic coronavirus strain, SARS-CoV, was the causative agent of the Middle East respiratory syndrome (MERS) outbreak in 2012, which had spread to 27 countries, with 858 known deaths since then (https://www.who.int/health-topics/middle-east-respiratory-syndrome-coronavirus-mers#tab=tab_1). Because SARS-CoV-2 is closely related to these strains, it is also likely to have originated from bats (5). COVID-19 has affected the world more negatively than either SARS or MERS because of its high contagiousness, which is estimated to be 2- to 3-fold higher than that of influenza (6). The case-fatality rates of COVID-19 vary widely in different countries, ranging from 1% to 15%; however, the rate typically lies between 2% and 4% (https://ourworldindata.org/coronavirus).

## COVID-19 PATHOGENESIS

SARS-CoV-2 infects primarily the respiratory system and may cause various symptoms, ranging from mild illness to significant hypoxia due to acute respiratory distress syndrome (7). Common COVID-19 symptoms include fever, cough, myalgia, fatigue, and pneumonia (2, 7). Diarrhea, nausea, and vomiting have also been reported, indicating that the gastrointestinal (GI) tract is also a site of infection (8–12). A substantial proportion of patients appear to have detectable GI symptoms, though this proportion varies depending on the different patient groups studied (9, 13). A recent modeling study using large data sets of reported cases suggested that SARS-CoV-2-infected patients first develop a fever and then respiratory symptoms, followed by GI tract symptoms, if they ever occur (14).

The virus uses its spike (S) protein to interact with angiotensin-converting enzyme 2 (ACE2), which is present on the surface of the epithelial cells lining the organs, including the lungs and GI tract (10, 15–17). Once the virus binds to ACE2, the type 2 transmembrane serine protease present in the host cell promotes viral uptake by cleaving ACE2 and activating the viral S protein, which mediates the entry of the virus into host cells (16). Next, the viral RNA genome enters the nucleus for replication. Viral reproduction kills the host cell, ultimately damaging the surrounding tissues as the cell destruction spreads.

Epithelial cells, alveolar macrophages, and dendritic cells are the main components of innate immunity in the airway (18). Dendritic cells residing underneath the epithelial cells and alveolar macrophages are the first to respond to viruses. Additionally, cellular damage in the lungs can lead to the release of the cytokines interleukin 8 (IL-8) and IL-6 by epithelial cells (19). As IL-8 acts as a chemoattractant to recruit neutrophils and T cells to the infection site, T cell responses are promptly initiated via antigen presentation by dendritic cells and macrophages. CD4^+^ T cells activate B cells to produce virus-specific antibodies, whereas CD8^+^ T cells kill the cells infected by the virus (20). In most cases, these local immune responses resolve viral infections. However, in some cases, the immune system is overwhelmed by viral damage. Conversely, the immune system may trigger a strong inflammatory cascade. Thus, in severe COVID-19 patients, the infiltration of numerous immune cells has been observed in the lungs (21), apart from the increased plasma concentrations of proinflammatory cytokines, including IL-6, IL-1β, and tumor necrosis factor alpha (7, 22). This phenomenon of abnormal cytokine overproduction is known as a “cytokine storm,” and it has been suggested as a cause of massive inflammation and tissue damage in patients, often leading to a serious outcome (23). However, the existence of this phenomenon in COVID-19 is currently controversial (24).

Apart from these direct effects, SARS-CoV-2 can indirectly damage the host by inhibiting the regular enzymatic function of ACE2 by binding to it. For example, altered ACE2 functionality in the lungs may contribute to the pathophysiological process of virus-induced acute lung injury (25). The expression of ACE2 in gut enterocytes is also an important regulator of dietary amino acid homeostasis, innate immunity, gut microbial ecology, and susceptibility to colitis; therefore, its inhibition can cause intestinal inflammation (26).

**FIG 1:**
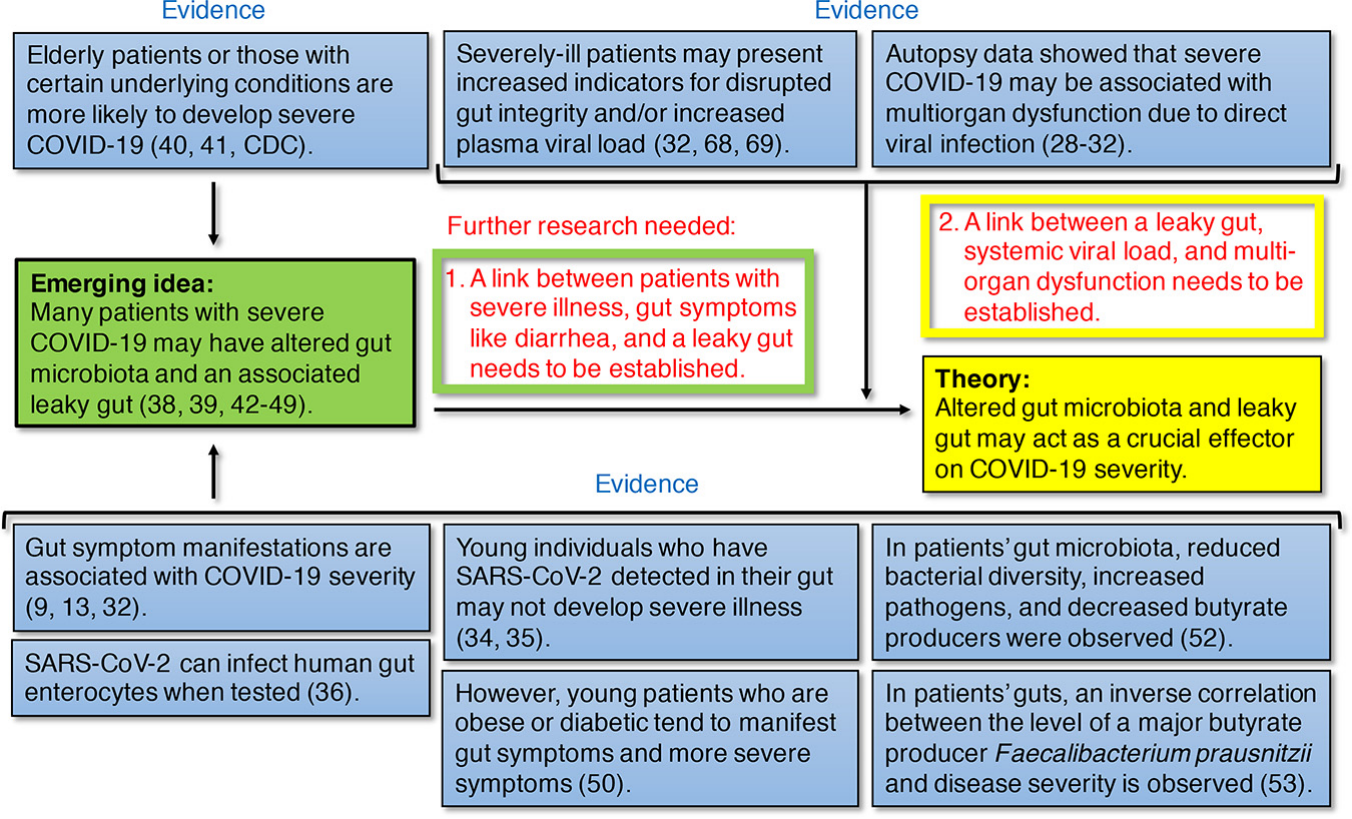
Lines of evidence supporting the hypothesis that a leaky gut affects COVID-19 severity and further studies that are needed. Current evidence supporting the emerging idea (in the green box) and evidence directly supporting the hypothesis (in the yellow box) are shown in the blue boxes with reference numbers. The ideas that are needed to be established through further research to support the emerging idea and the hypothesis are highlighted in matching green and yellow boxes, respectively.

While postmortem examination is invaluable in dissecting details of COVID-19 pathology, little has been reported to date. However, it is now clear that diffuse alveolar damage with capillary congestion and necrosis of pneumocytes, along with various additional features, is the major manifestation in the lung (27). Intriguingly, data also showed that COVID-19 causes extrapulmonary manifestations in various organs, including the GI tract, liver, kidney, heart, spleen, brain, and bone marrow, with occasional traces of viral infection (Fig. 1) (28–30). Moreover, in an autopsy series of 22 patients focusing on the bodily distribution of SARS-CoV-2, the researchers were able to detect and quantify viral loads in multiple organs, including the lungs, pharynx, heart, liver, brain, and kidneys in most dead patients (31). The kidneys had the highest viral load among non-respiratory tract organs examined, even in patients without a history of kidney disease (31). Although more extensive studies are needed, these findings suggest that extrapulmonary multiorgan dysfunction may be associated with severe illness in patients with COVID-19, and it is likely caused by direct exposure to SARS-CoV-2. Consistently, a recent study showed that a high plasma load of SARS-CoV-2 is associated with increased disease severity and risk of mortality (32).

## ALTERED GUT MICROBIOTA MAY LEAD TO SEVERE COVID-19 SYMPTOMS

A substantial proportion of hospitalized patients with respiratory symptoms also have GI symptoms, such as diarrhea, nausea, and vomiting (8–12, 33). Furthermore, seriously affected patients tend to present SARS-CoV-2 in the GI tissues or have GI symptoms, suggesting that the involvement of this virus in the GI tract increases disease severity (Fig. 1) (9, 13, 33). Nevertheless, the presence of SARS-CoV-2 in the GI tract may not always lead to GI symptoms (Fig. 1). For instance, in a study conducted in Singapore, 50% of the examined COVID-19 patients had a detectable level of virus in their feces, but only half of them showed GI symptoms, such as diarrhea (34). In a study of 12 young COVID-19 patients under 18 years of age (3 asymptomatic and 9 with mild symptoms), the virus was detected in patient feces at higher and longer-lasting levels than in nasopharyngeal samples (35). In fact, although SARS-CoV-2 is capable of infecting human gut enterocytes when tested on human small intestinal organoids (Fig. 1) (36), it may differ in an actual healthy gut. This stems from the multiple defense systems that protect it, including a thick mucus layer (∼700 μm) (37), colonization resistance conferred by the gut microbiome (38), an epithelial layer with tight junctions, and numerous host factors, such as immunoglobulin A, proteases, and peptides with protective and antimicrobial functions (37, 39).

**FIG 2:**
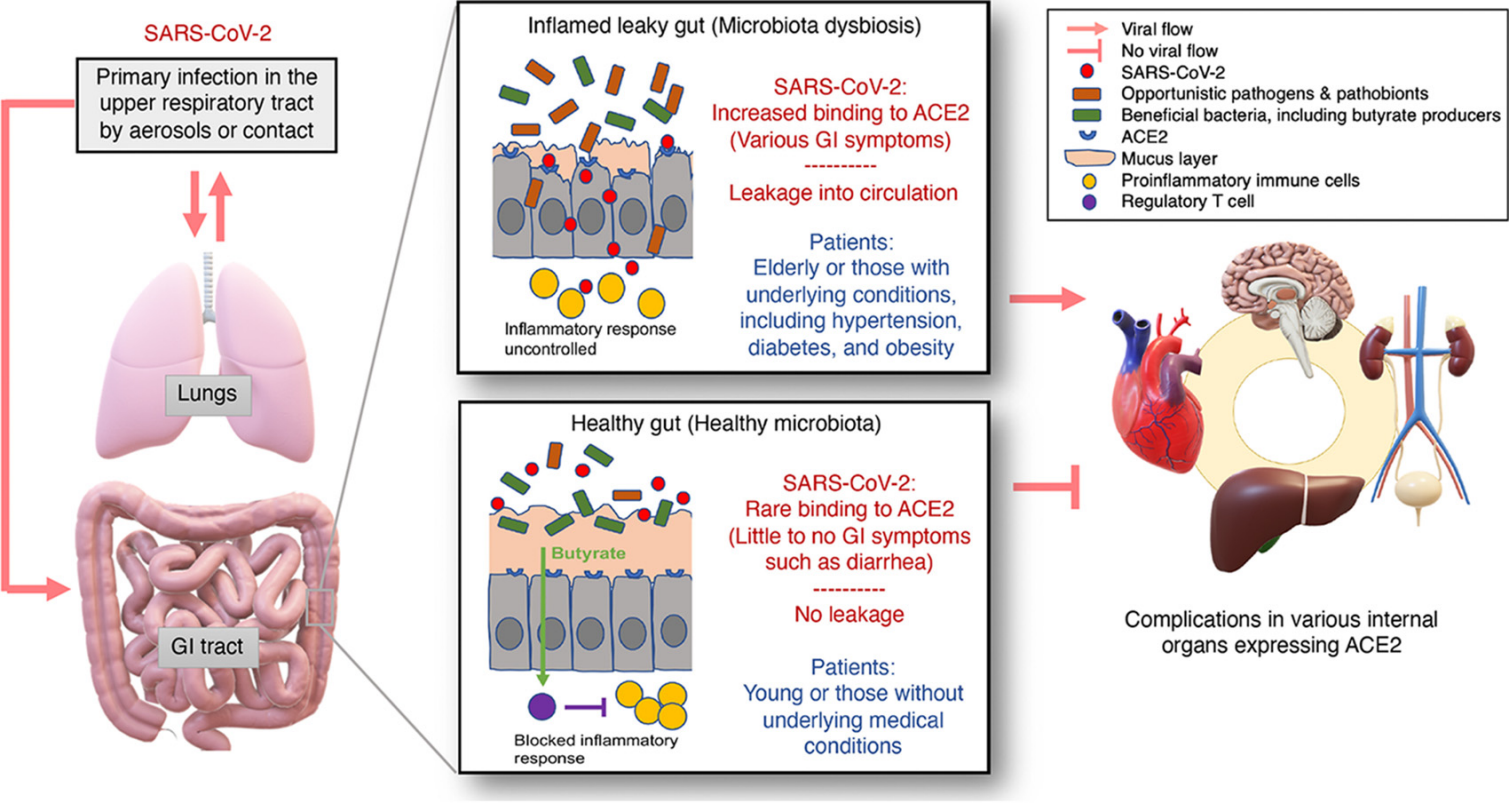
Model for COVID-19 pathogenesis leading to extrapulmonary complications. Localized infections by SARS-CoV-2 mostly begin in the respiratory system and then progress to the GI tract; they may later develop into a systemic disease, resulting in multiorgan complications. Disrupted gut barrier integrity associated with elderliness or underlying chronic conditions, such as hypertension, diabetes, and obesity, may be a crucial effector that allows the virus to gain access to ACE2 on the enterocytes and leak out of the GI tract to spread throughout the body. If SARS-CoV-2 penetrates the gut barrier, it may cause inflammation due to overly reactive immune responses that thereby further increase its leakage from the gut. Contrastingly, in a healthy GI tract with a higher number of T_reg_ cells due to their activation by butyrate, such as in young healthy children, the virus may be contained in the GI tract and excreted in feces without posing a considerable threat to the other organs of the body.

Because SARS-CoV-2 can be prevalent in the GI tract regardless of the presence of symptoms, gut health at the time of infection may be critical for symptom development. Elderly patients or those with certain underlying medical conditions, such as high blood pressure, diabetes, and obesity, are highly vulnerable to the disease (Fig. 1 and 2) (40, 41; https://www.cdc.gov/coronavirus/2019-ncov/need-extra-precautions/people-at-increased-risk.html). Both elderliness and chronic conditions may be associated with an altered gut microbiota that affects gut barrier integrity, such that pathogens and pathobionts gain more access to the surface of the enterocytes (Fig. 1 and 2) (38, 39, 42–49). Even among younger individuals, who are typically less likely to develop symptomatic COVID-19, patients with obesity or diabetes tend to manifest more severe symptoms, suggesting that the presence of chronic conditions has a stronger effect than younger age (Fig. 1) (50). As elderly people are generally patients with chronic diseases, they can be highly vulnerable to COVID-19 (51).

In a recent study, Gu et al. showed a significant reduction in bacterial diversity in gut microbiota samples collected from COVID-19 patients compared with those obtained from healthy controls (Fig. 1) (52). Additionally, they observed an enrichment of opportunistic pathogens and depletion in the abundance of beneficial bacteria, including those belonging to the *Ruminococcaceae* and *Lachnospiraceae* families. Such changes in the gut microbiota are generally considered typical signs of dysbiosis and an unhealthy gut (44–46). Moreover, the researchers found that influenza A (H1N1) infection reduced gut microbiome diversity in patients but resulted in an overall microbial composition different from that in COVID-19 patients (52). In another study, Zuo et al. reported significant gut microbiome alterations in COVID-19 patients (53), and they found an inverse correlation between the abundance of the beneficial gut species Faecalibacterium prausnitzii and disease severity (Fig. 1). Notably, they found the abundance of a few *Bacteroides* spp., which downregulate the expression of ACE2 in the murine gut, to be inversely correlated with SARS-CoV-2 load in patient fecal samples (53). This finding emphasizes the importance of the interrelationship between the gut microbiome, ACE2 expression, and viral infection. In another study that reported a strong correlation between COVID-19 risk and an altered gut microbiota (54), Gou et al. argued that unhealthy gut microbiomes may be the underlying reason for the predisposition of normal individuals to severe COVID-19 (54).

Notably, these studies demonstrating a close link between gut microbiota dysbiosis and COVID-19 severity have reported a common finding (52–54). Beneficial bacteria, whose abundance was reduced in COVID-19 patients, were reported to belong to the families *Ruminococcaceae* or *Lachnospiraceae* (52), a single species *F. prausnitzii* (53), and the class *Clostridia* (54). The class *Clostridia* includes the family *Ruminococcaceae*, which includes the species F. prausnitzii, which is one of the major butyric acid-producing bacteria in the gut (55). While the beneficial impact of *F. prausnitzii* on human health is well established (56), a subspecies that causes a predisposition to atopic dermatitis in infants and young children by competing with the beneficial members of the species has been identified (57). The intricate microbial interactions and the physiology involving this important butyrate-producing species warrant future investigations to understand its influence on human health and disease (57, 58).

Butyric acid, produced by many beneficial gut bacteria belonging to *Clostridia*, is a short-chain fatty acid (SCFA), which, along with propionic and acetic acids, is a fermentation product of dietary fiber that plays a pivotal role in gut health (Fig. 2). It helps maintain gut barrier integrity by serving as an important energy source for colonocytes, inhibiting the activation of NF-κB, activating the G protein-coupled receptor pair of GPR41 and GPR43, inhibiting histone deacetylase activity, which causes anti-inflammatory activities, and promoting regulatory T cells (T_reg_) cells (59–64). T_reg_ cells have a central role in the suppression of inflammatory and allergic responses (Fig. 2) (59). Depletion of certain butyric acid producers in the gut microbiota has been identified in a few chronic diseases, including allergies, inflammatory diseases, colorectal cancer, and Crohn’s disease (56, 57, 65).

## DISRUPTED GUT BARRIER INTEGRITY MAY BE ATTRIBUTABLE TO EXTRAPULMONARY COMPLICATIONS, INCLUDING HEART, LIVER, KIDNEY, AND BRAIN DYSFUNCTION

Along with having respiratory and GI symptoms, COVID-19 patients often manifest other symptoms, such as headache and hepatic, pancreatic, and cardiac dysfunction (66). This coincides with the fact that ACE2, the receptor for SARS-CoV-2, is expressed not only in the lungs and the GI tract but also in various other organs, including the liver, heart, kidneys, bladder, and brain (19, 25, 29). Since ACE2 regulates vital processes, such as normal cardiac function (e.g., blood pressure control), optimal beta-cell function, and insulin sensitivity (25), if these organs are damaged or their essential ACE2 functions are blocked by the virus, various complications may occur (67). Histopathological evidence for viral infection and/or actual viral components has been observed in various internal organs in biopsy samples from severely ill patients or from autopsy specimens (28–31).

Thus, it is important to determine how SARS-CoV-2 reaches internal organs other than the lungs or GI tract. Altered gut microbiotas, which may be associated with elderliness and certain underlying medical conditions that predispose COVID-19 patients to severe symptoms, often lead to disrupted gut barrier integrity (Fig. 1 and 2) (42–49). While a link between patients with severe illness, gut symptoms (e.g., diarrhea), and a leaky gut still needs to be established (Fig. 1), it is highly likely to be the case because patients with diarrhea present increased levels of systemic IL-6 and fecal calprotectin, which are indicators of gut inflammation and disrupted gut integrity, such as a thinned mucus layer and reduced tight junctions between enterocytes (Fig. 2) (68). Drastically elevated plasma IL-6 concentrations have been associated with the presence of SARS-CoV-2 RNA in the plasma of critically ill patients (32, 69). It is therefore plausible that critically ill COVID-19 patients may have a disrupted gut barrier, also known as a “leaky gut” (47–49, 70), which may allow SARS-CoV-2 to not only bind to ACE2 on the enterocytes but also exit the GI tract and enter the bloodstream, allowing it to access various organs expressing ACE2 throughout the body (Fig. 2). If SARS-CoV-2 penetrates the gut barrier, it may cause inflammation due to overly reactive immune responses, thereby further increasing gut leakage (Fig. 2) (59). Contrastingly, in a healthy GI tract with a high number of T_reg_ cells that are activated by butyrate, such a proinflammatory response may be blocked (59). However, a link between a leaky gut, plasma viral load, and extrapulmonary multiorgan dysfunction remains to be established (Fig. 1).

## CONCLUSIONS AND PERSPECTIVES

**FIG 3:**
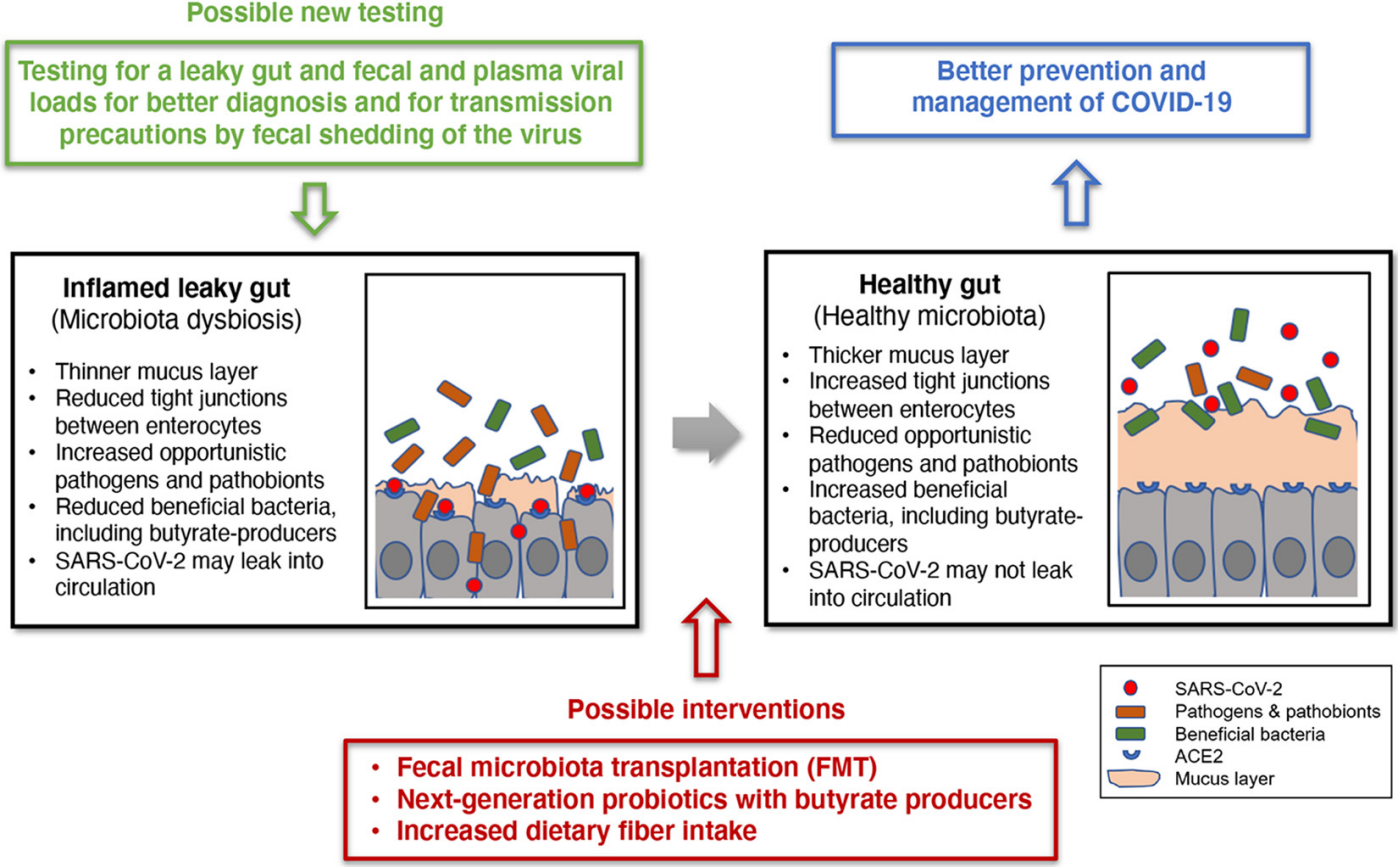
Exploiting the gut microbiota for better COVID-19 disease prevention and management. Testing for leaky gut and fecal and plasma viral loads may be used to improve diagnoses for seriously ill patients and for establishing a basis for transmission precautions from some patients who may have prolonged fecal shedding of the virus even after viral clearance in the respiratory tract. This presents the intriguing, but presently unsubstantiated, possibility that an inflamed leaky gut, which may be associated with a higher risk of severe illness, may be improved or treated via a few interventions. FMT, production of next-generation probiotics focusing on butyrate-producing gut microbes, or simply increasing the daily intake of dietary fiber may be considered in improving the gut health of COVID-19 patients.

A strong pattern has emerged from patients with severe COVID-19, as many of them are either elderly or have certain underlying medical conditions which may be associated with an altered gut microbiota (38, 39, 42–49). Such dysbiosis of the gut microbiota may be associated with disrupted gut barrier integrity, which may allow SARS-CoV-2 to gain access to the otherwise well-protected enterocytes and to circulate and infect internal organs expressing ACE2 (Fig. 2). If this is what is happening in the serious cases of this illness that present extrapulmonary multiorgan dysfunction, testing for a leaky gut and fecal and plasma viral loads will be of high value for a more accurate prognosis, particularly for those likely to have altered gut microbiotas (Fig. 3). Furthermore, fecal viral load data can also be useful for informing transmission precautions, because some patients may have prolonged fecal shedding of the virus even after viral clearance in the respiratory tract (Fig. 3) (33, 35).

While developing treatments and vaccines for COVID-19 is of prime significance, exploiting the gut microbiota to improve disease prevention and management may also be important (Fig. 3). The first treatment to be considered for the seriously ill may be fecal microbiota transplantation (FMT). This practice enables stool infusion from a healthy individual to a patient with presumed gut microbial dysbiosis (71). FMT has been remarkably successful in the treatment of Clostridioides difficile infection (CDI) (72). Although FMT has shown only marginal success in treating other conditions, such as inflammatory bowel disease and metabolic disorders, COVID-19 may not be the same because, unlike these inflammatory disorders, it is an infectious disease as CDI, which has a clear and simpler therapeutic target. However, in any case, safety issues associated with carrying over undetected additional potential pathogens need to be seriously considered before FMT can be explored in the context of COVID-19 (72). The development of next-generation probiotics focusing on butyrate-producing gut microbes can also be pursued (73). However, these novel microbial therapeutics still need to overcome the hurdles of the regulatory framework (73). Lastly, simply increasing the daily intake of dietary fiber may markedly help improve gut health (74), as fiber is directly utilized by beneficial gut microbes to produce SCFAs, with butyrate being a key substance (74). This dietary adaptation may be the most easy and effective method that can be considered to be implemented immediately to prevent severe COVID-19 or just for general health improvement.
